# A Tooth Drawn by a Hen

**Published:** 1891-02

**Authors:** 


### Editorial, Etc.

A TOOTH DRAWN BY A HEN.—Virginia is thus far the only known state which boasts of hen dentists. Some time since the *Page County Courier* published an account of a hen there angrily flying at a farmer and pulling out one of his teeth. George E. Heath, who lives in Hanover county, a short distance from Ashland, was in the city. He states that some time ago he went to his hennery and attempted to take a hen off her roost, when she flew in his face, pecked him in his mouth, and took out a tooth which had been troubling Mr. Heath for some time, and which he intended having extracted. This is the second hen dentist in Virginia.

